# She Asked for It? Descriptions of Victims' Behaviors Are Associated With Sentencing in Norwegian Rape Trials

**DOI:** 10.1111/sjop.13088

**Published:** 2024-12-15

**Authors:** Kirsten Rønning Rinde, Tea Vučić, Maria Grønland Andresen, Audun Havnen, Stian Solem

**Affiliations:** ^1^ Department of Psychology Norwegian University of Science and Technology Trondheim Norway; ^2^ Independent researcher; ^3^ Faculty of Medicine University of Oslo Oslo Norway

**Keywords:** court decisions, rape myths, sexual violence

## Abstract

Feminist theorists have long argued that rape myths contribute to normalizing sexual assault, through belittling and denying rape victims' claims. This study examines whether descriptions of victims' behaviors are associated with sentencing in rape trials. A total of 2054 Norwegian court decisions from 2013 to 2023 in judicial records were screened. Fifty‐one of these included descriptions of the victims' behavior as operationalized by a subscale of the Illinois Rape Myth Acceptance Scale called “She Asked For It” (IRMAS‐SAFI; type of clothing, going to a room alone with a guy at a party, previous sexual behavior, saying no unclearly, and kissing). Matching cases without such descriptions were then selected, resulting in a total sample of 102 court decisions. In addition, a randomly selected comparison group (*n* = 51) was included for robustness analysis. Results revealed that defendants who had attacked a victim in the IRMAS‐SAFI group were sentenced to fewer months in prison (*M* = 25.3, SD = 20.9) than defendants from the comparison group (*M* = 41.7, SD = 13.3). This type of description of victims' behavior was significantly associated with shorter prison sentences when controlling for medical evidence, age of the defendant, and use of violence. The results indicate that implementing measures to reduce the influence of rape myths on judges' evaluations in rape trials could lead to fairer court decisions.

**Summary**

- Are descriptions of victims' behaviors in court verdicts associated with sentencing in rape trials?
- The study coded descriptions of behaviors such as type of clothing and previous sexual behavior.
- Verdicts with such descriptions were associated with fewer months in prison.
- Rape myths could influence sentencing in rape trials.

Rape is a common problem in most societies. A Norwegian survey from 2023 indicated that one in five Norwegian women has been raped at least once in their lifetime (Dale et al. 2023). Less than half of the female participants had contacted health professionals after the assault, and only 20% had contacted the police (Dale et al. 2023). The results coincided with earlier Norwegian research, which has generally shown that rape is a prevalent and underreported crime (Løvgren, Høgestøl, and Kotsadam 2022). In addition, a large portion of reported rapes never reach the court. In 2022, 61% of reported rape cases in Norway were dismissed due to missing evidence or police lacking capacity (Statistics Norway 2023). Research from other countries have found similar results (McQueen and Kelly 2021; Wolitzky‐Taylor et al. 2011).

The low reporting‐ and conviction rates of sexual assault are worrying, considering the grave consequences for those victimized. A North American meta‐analysis from 2020 found that 36% of people who had been sexually assaulted met the criteria for life‐long posttraumatic stress disorder, compared to 9% of people who had not been sexually assaulted (Dworkin 2020). The prevalence of other disorders such as anxiety, depression, eating disorders, and substance abuse was also higher among sexual assault survivors (Dworkin 2020). Such disorders can reduce victims' ability to perform in everyday life, for example in educational and professional settings, and cause difficulties in intimate relationships. These consequences are detrimental to survivors and costly to society, emphasizing the importance of effectively responding to sexual violence.

Since the 1970s, feminist theorists (i.e., belief in and advocacy of political, economic, social, and gender equality) have argued that common stereotypical beliefs about rape are an important contributor to the low reporting rates of rape, and to aggravating the psychological trauma experienced by victims (Brownmiller 1975). These beliefs, called *rape myths*, can be defined as stereotypical and false beliefs about rape, rapists, and rape victims that deny, belittle, and justify rape (Burt 1980; Lonsway and Fitzgerald 1994). Rape myths include false beliefs about how the actions of a victim before, during, or after a sexual assault relate to credibility and consent. Belief in rape myths is strongly related to endorsement of traditional gender roles and acceptance for interpersonal violence (Burt 1980; Payne, Lonsway, and Fitzgerald 1999; Angelone, Mitchell, and Grossi 2015). Rape myth *acceptance* is the acceptance of false beliefs, stereotypes, and statements about rape, victims, and perpetrators (Burt 1980). Studies from Canada, the United States, and the United Kingdom have shown that these false beliefs are held by a significant proportion of the public, including health professionals, students, and police (Suarez and Gadalla 2010; McMahon and Farmer 2011; Hine and Murphy 2017). As an example, around 20% of US police officers agreed that provocatively dressed women invite sex, and that any healthy woman can resist rape if they really want to (Page 2007). One British study indicated that rape myth acceptance is also prevalent among the victims themselves (Egan and Wilson 2012), and a North American study discovered that victims who believe in rape myths are less likely to report, either because they are unsure whether the assault was rape or because they blame themselves for provoking the attack (Peterson and Muehlenhard 2004).

The Rape Myth Acceptance Scale (RMAS; Burt 1980) consists of 19 items, such as “In the majority of rapes, the victim is promiscuous or has a bad reputation” and “If a girl engages in necking or petting and she lets things get out of hand, it is her own fault if her partner forces sex on her.” Both statements measure the belief that a woman who behaves promiscuously or recklessly must blame herself if she is raped. The RMAS was widely used in research but later replaced by the Illinois Rape Myth Acceptance Scale (IRMAS) due to psychometric issues and outdated language (Payne, Lonsway, and Fitzgerald 1999). The IRMAS has also been updated and revised several times (McMahon and Farmer 2011; Thelan and Meadows 2022).

Research using various versions of IRMAS indicates that acceptance of rape myths has declined since the measure was first published (McMahon and Farmer 2011; Egan and Wilson 2012; Bendixen, Henriksen, and Nøstdahl 2014). Increased gender equality and decreasing levels of sexism could be an important contributor to this decline (Huang, Osborne, and Sibley 2019; Chon and Clifford 2020). Levels of gender equality and sexism vary across nations and cultures, and Norway is often viewed as one of the world's most gender‐egalitarian countries (Gornick and Meyers 2008). Some have argued the existence of a “Nordic pattern” in which women in Nordic countries show more sexual agency than women in other nations, and that this is a sign of gender equality (Kraft 1991). However, traditional gender role stereotypes and double standards exist in Norway and in other countries, and Norwegian women are as exposed to sexual violence as women in other northern European countries (Lukasse et al. 2014; Tutenges, Sandberg, and Pedersen 2020). One possible explanation for declining acceptance of rape myths is that the decrease may be caused by social desirability effects, rather than an actual decline (Thelan and Meadows 2022). One US study argued that as awareness of the negative effects of rape myths has increased, holding such beliefs has become unpopular. This could lead to the expression of rape myth acceptance being more subtle, and thus harder to measure (Thelan and Meadows 2022).

It is also possible that the decrease might be due to a change in which rape myths are believed. When testing a short version of the IRMAS using a Norwegian sample, researchers found that participants were most supportive of rape myths such as “Any woman who teases a man sexually and doesn't finish what she started realistically deserves anything she gets” or “When a woman allows petting to get to a certain point, she is implicitly agreeing to have sex” (Bendixen, Henriksen, and Nøstdahl 2014). The participants were less in agreement with statements such as “Women tend to exaggerate how much rape affects them” or “A rape probably didn't happen if the woman has no bruises or marks” (Bendixen, Henriksen, and Nøstdahl 2014).

In a different study, the same short version of the IRMAS was used in a survey of Norwegian lay and professional judges (Bendixen et al. 2014). The survey showed low acceptance for rape myths among the 243 respondents, with agreement rates between 0% and 11.1%. The participants were most in agreement with items targeting the belief that evaluating the victim's past and character is important in rape cases (11.1%), that rape happens because men can't control themselves (9.2%), and that women who behave promiscuously signal willingness to have sex and are thus partly responsible if they are raped (6.4%) (Bendixen et al. 2014). The results from these two studies could indicate that the decrease in rape myth acceptance is specific to some of the categories included in the IRMAS, making some myths more relevant than others.

In court trials, the judges' discretionary evaluation of the victim and defendant's credibility is crucial for the conviction and sentencing. This is especially true for rape trials, in which there are often few witnesses and little available evidence. The victim's court testimony is, however, sufficient for conviction, if this testimony is credible and cohesive. If the judges' evaluation of the victim and the credibility of her explanation are based on stereotypical expectations of a rape victim, this can lead to unfair court decisions.

Some studies conducted in the United Kingdom, United States, and Australia have indirectly explored the effect of rape myths on the evaluation of guilt and sentencing in rape cases (Hine and Murphy 2017; Ryan and Westera 2018; Klement, Sagarin, and Skowronski 2019; Leverick 2020; Berkland, Ji, and Jain 2022). One US study investigated whether using different labels for sexual assault affected participants' evaluation of the assault (Berkland, Ji, and Jain 2022). The study was conducted using a sample of 152 students split into three groups. The groups were given a hypothetical rape scenario, with the only difference between the groups being the labels used: “rape,” “sexual assault,” or “non‐consensual sexual intercourse.” Results showed that participants who read the labels “rape” and “sexual assault” were less inclined to perceive the perpetrator as guilty compared to the participants who read “non‐consensual sexual intercourse.” The authors proposed that this effect could be caused by the first two terms being more effective in eliciting rape myths and gender stereotypes, encouraging a specific perception of the scenario.

Another study explored how rape myths may influence evaluation and decision‐making in Australian rape trials (Ryan and Westera 2018). The authors tested the effect of having an expert witness testimony and a cognitive statement from the complainant, which is the complainant's own explanation of why she reacted to the assault in the way she did, on the participants' perception of a trial testimony. An expert witness testimony includes information that explains counterintuitive behavior of rape complainants, such as delayed reporting. The 280 participants found the defendant more blameworthy when the expert witness testimony was present, and the participants used more evidence‐based reasoning when both the expert testimony and the complainant cognitive statement were included (Ryan and Westera 2018). The authors argued that this effect may be caused by the cognitive statement and the expert testimony “filling in” missing information, which would otherwise be filled in by the participants own assumptions.

These studies provide valuable insight into the ways in which rape myths can influence attribution of blame and responsibility in rape cases. The studies do, however, have some limitations as they used student samples and vignettes. This means that the results may not be transferable to real court trials. Some qualitative studies from the United Kingdom, Norway, and Sweden have explored how rape myths and gender stereotypes may influence sentencing and verdicts in rape trials by analyzing written court decisions (Bitsch 2019; Laugerud 2020; Smith and Skinner 2017; Wallin et al. 2021). In one of these, the author conducted a discourse analysis of justifications for court decisions from the Norwegian Court of Appeal and argued that how the victims met normative expectations of gender, sexuality, and the body (e.g., size of the victim) influenced whether an incident was perceived as rape or consensual sex (Laugerud 2020). One of the cases described in the study featured a female security guard, who reported her male colleague for rape. The defendant was acquitted on the basis that the victim was a trained security guard, weighing 80 kg, and should therefore have been able to stop the assault. In this example, the author concluded that the victim was perceived as abnormally masculine, and thus deviated from judges' expectations of a rape victim. The study pointed out that rape myths are a part of what we call common sense, a type of knowledge that is rarely consciously reflected over. If this is true, rape myths could be expected to have a larger influence on court decisions than what is indicated by survey‐based studies using IRMAS. Because the study was qualitative, it is uncertain whether the cases described can be said to reflect a tendency in Norwegian courts, or if they are exceptions.

Therefore, this study will explore whether rape myths are associated with sentencing in Norwegian rape trials. Based on the two studies conducted on Norwegian samples, it is assumed that the category of rape myths regarding the IRMAS subscale She Asked For It (IRMAS‐SAFI) will be the most relevant for this purpose. The aim of this study is to investigate whether descriptions of such behavior is associated with the sentence the defendant received. It was hypothesized that defendants in rape trials receive more lenient sentences when such descriptions of the victim's behaviors are included.

## 1 Method

This study was conducted using a quasi‐experimental design. To discover whether defendants received more lenient sentences according to descriptions of victims' behaviors, two groups of court decisions were compared. One group consisted of decisions from rape cases that included descriptions matching the descriptive elements (not normative) of the Illinois Rape Myth Acceptance Scale—“She Asked For It” (IRMAS‐SAFI) items, and the second group of decisions from cases that did not include such a description. The groups were compared on the severity of the sentence defendants received. This was measured by months in prison and financial penalty in NOK (Norwegian kroner). In addition, information about what type of rape the case concerned, how severe the rape was, what evidence was available, the age of the defendant, and what year the verdict was pronounced, was also included in the dataset.

### 1.1 Data Selection

The data used in this project were collected from court decisions sourced from Lovdata (Lovdata.no). Lovdata is a database created by the Norwegian Department of Justice and the Faculty of Law at the University of Oslo, which contains legal information such as Norwegian laws and regulations, legal articles, and court decisions. To find relevant court decisions, Lovdata was searched using the following Norwegian terms related to rape: “overfallsvoldtekt,” “overgrep,” “utuktig,” “valdtekt,” “voldta,” “voldtekt,” and “voldtok.” The search conducted was limited to trials from 2013 to 2023, and all cases were sexual offense cases from the Court of Appeal (Lagmannsretten). In addition to the abovementioned delimitations, all cases involving children, incest, domestic abuse, and individuals with intellectual disabilities or mental illnesses were excluded. Cases involving multiple perpetrators or multiple victims were also excluded. This was done because cases where these factors are present carry a different sentencing, and often include more than one indictment. Additionally, cases involving additional sentencing for defendants convicted of previous offenses were excluded. Cases concerning male victims were also excluded, because it is assumed that judges' evaluation of rape victim credibility is affected by different expectations and assumptions for men and women.

**FIGURE 1:**
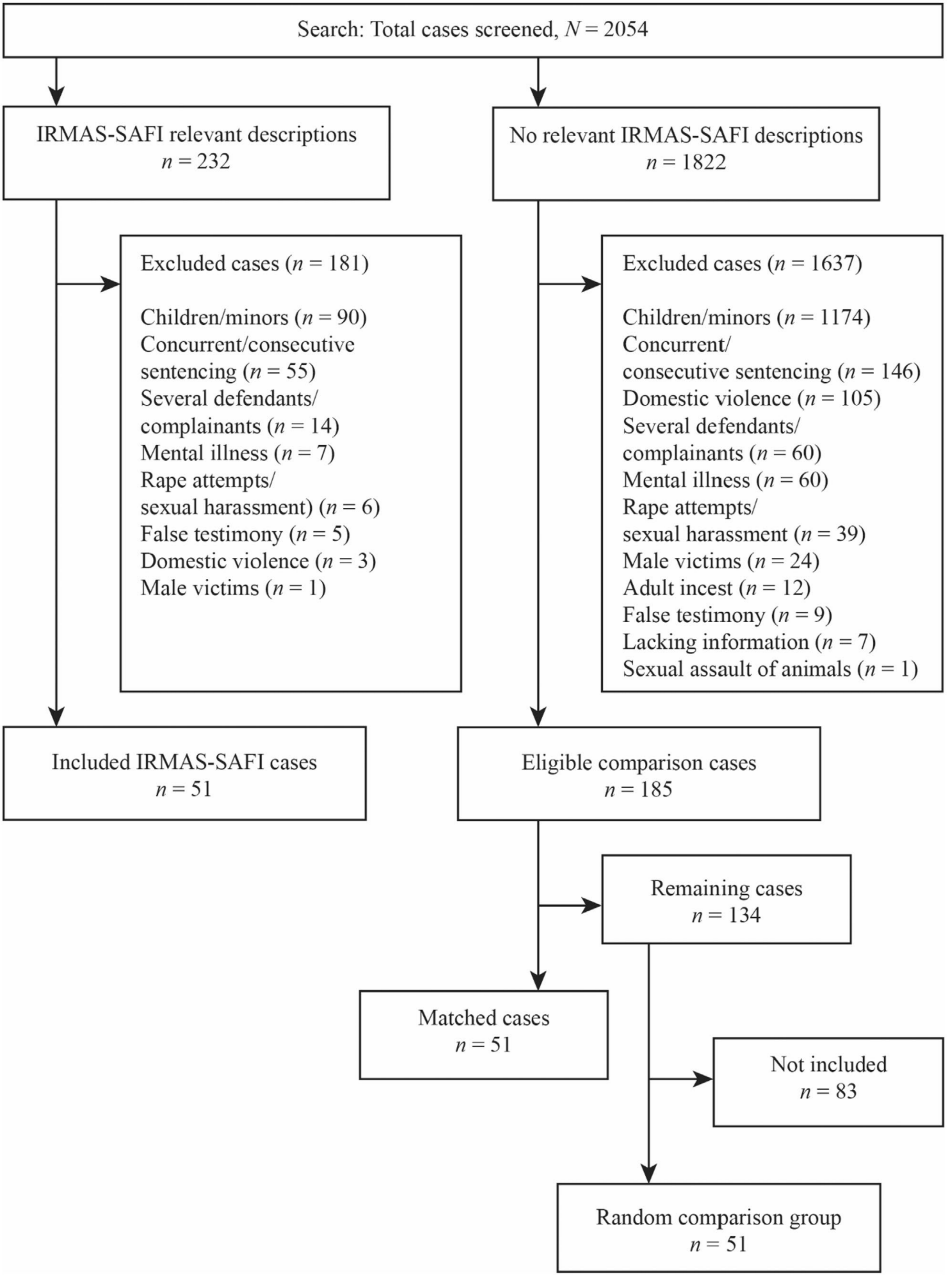
Flowchart summarizing the screening and selection process.

To decide which cases would be selected for the analytic sample, the IRMAS was used (McMahon and Farmer 2011). In addition, a similar number of comparable cases without any IRMAS content were selected for a matched comparison group (see Figure 1 for a detailed description of the selection process). The IRMAS consists of 22 statements, divided into four categories: “She Asked For It,” “He didn't mean to,” “It wasn't really rape,” and “She lied.” This study used the first of these categories (IRMAS‐SAFI). The items included in the “She Asked For It” category state that promiscuous or reckless behavior makes the victim partly responsible if she is raped. Five of the items were used to categorize cases as including descriptions of victims' behavior as matching with the IRMAS‐SAFI or not. Only the descriptive parts of the IRMAS‐SAFI items were used (underlined text) and not the normative/evaluative part: “When girls go to parties wearing slutty clothes, they are asking for trouble,” “If a girl goes to a room alone with a guy at a party, it is her own fault if she is raped,” “If a girl acts like a slut, eventually she is going to get into trouble,” “When girls are raped, it's often because the way they said “no” was unclear,” and “If a girl initiates kissing or hooking up, she should not be surprised if a guy assumes she wants to have sex” (McMahon and Farmer 2011). The last statement was understood as including instances of the victim engaging in consensual kissing or casual sex with the perpetrator prior to the assault. Engaging in sexual activities with a partner while being in a romantic relationship was not classified as a description of IRMAS‐SAFI behavior.

In many rape trials, the defendant claims that the complainant had initiated kissing or participated willingly in the sexual activity. Therefore, the descriptions of IRMAS‐SAFI behavior had to come from another source than the defendant's explanation to be included in the IRMAS‐SAFI group. This means that information was not included if the court decision specified that it was only said by the defendant. Additionally, the item stating that the victim is responsible if she is raped after going to a room with a guy at a party was interpreted as not including instances where the victim had joined the perpetrator for a stated purpose, such as fetching something, using the bathroom, or vomiting.

The “She Asked For It” category also includes a sixth statement, “If a girl is raped while she is drunk, she is at least somewhat responsible for letting things get out of control.” This item was excluded for the purpose of this study because intoxication among victims is very common in rape cases (Jones, Holmgren, and Ahlner 2012; Mohler‐Kuo et al. 2004).

Two independent raters were given two different tasks to check for subjective bias in the selection process. The first screened court decisions from 1 year and saved all cases deemed to fit the criteria described above. The second rater read all court decisions included in the dataset and categorized the decisions into two groups, one including descriptions of IRMAS‐SAFI victims and one without. The rater also categorized the decisions into the five different IRMAS statements. To minimize the chance of bias, they were only given the selection criteria as described above and had no knowledge of the hypothesis. When there were discrepancies in the selections made by the author and the independent raters, a consensus was reached by discussion of the court decisions considering the inclusion criteria.

When all cases were screened, and the cases containing descriptions of IRMAS‐SAFI behavior were found, matching cases without such descriptions were collected to be used as a comparison group. These cases were chosen so that the matching pairs concerned the same type of rape. Four categories of rape were used: Incapacitated rape without intercourse, incapacitated rape including intercourse, forceful rape without intercourse, and forceful rape including intercourse. The matching cases were also chosen to have similar levels of severity. Severity was measured by the amount of violence used, ranging from no additional violence used to excessive violence. Information about where the rape took place was also used to compare severity, because it is sometimes considered aggravating if a rape for instance happened in a public place (Bitsch and Klemetsen 2017).

To make sure the matching cases had similar levels of evidence available, information about how many witnesses were present to testify, whether medical evidence had been secured, whether there was any evidence in the form of text messages or phone calls logged, and if there were any photograph or video evidence to support the complainant's case, was extracted from each court decision. Because research has consistently shown that men tend to score higher than women on rape myth acceptance scales and are more likely to blame the victim in rape scenarios (Bendixen, Henriksen, and Nøstdahl 2014; McKimmie, Masser, and Bongiorno 2014; Thelan and Meadows 2022), the proportion of female judges present at each trial was also included in the dataset. The court usually consists of three court of appeal judges together with four lay judges. As far as it was possible, the matching cases were collected from the same year. For an overview of all court decisions and variables included in the dataset, see Table S1.

### 1.2 Statistical Analysis

To compare the IRMAS‐SAFI group and the comparison group on the continuous variables included in the dataset, a *t*‐test using the Bonferroni correction was used. The groups were compared on categorical variables, for example, type of rape, using a chi‐squared test.

The Shapiro–Wilk's test for normality was significant when used on the months in prison variable, indicating that the assumption of normality was not met. However, examination of the QQ‐plot showed an acceptable normal distribution. As the data were not clearly normally distributed, two types of tests were utilized to compare cases describing IRMAS‐SAFI behavior with those without on two dependent variables: financial penalty and prison sentence. Both the non‐parametric Mann–Whitney *U* test for independent samples and Welch's *t‐*test were conducted to determine the difference between the IRMAS‐SAFI group and the comparison group. The effect size was calculated using Cohen's *d*.

To investigate whether there were any confounding variables in the dataset, a linear regression analysis using forced entry was conducted. The regression model included the variables medical evidence, age of the defendant, place of rape, degree of violence, and IRMAS‐SAFI as predictors of months in prison. Possible existence and influence of rape myths were deducted based on whether IRMAS‐SAFI descriptions are associated with differences in sentencing.

## 2 Results

In total, 2054 court decisions were screened and 1818 of those were excluded. Following the five relevant IRMAS statements mentioned above, all remaining cases involving a description of the victim flirting, kissing, having consensual casual sex with others, or dressing provocatively were added to the dataset. For instance, many cases involved information about the victim having consensual, casual sex with other individuals prior to the reported rape. The screening and selection processes are illustrated in Figure 1.

Ultimately, 51 cases were found to fit the IRMAS‐SAFI criteria. Many of the cases collected could fit two or three of the five items used to categorize them, and these cases were therefore placed into several of the categories. Most of the cases described victims who had initiated kissing and other intimate activity prior to the assault, *n* = 39. The second most common category was the category called “a girl goes to a room alone with a guy at a party,” *n* = 30. Sixteen cases concerned victims engaging in casual, consensual sexual activities or kissing with other persons than the defendant prior to the assault. These cases were categorized under the statement “If a girl acts like a slut…”. Eight cases were found to fit the category concerning provocative clothing, and three were categorized under the statement “…the way they said “no” was unclear.” For a complete overview of how the written court decisions were interpreted and coded into the different IRMAS‐items, see supplemental Table S2.

The mean age of the defendants across the whole dataset was 30.56 (SD = 8.67), ranging from 20 to 62. On average the defendants received 33.45 (SD = 19.28) months in prison and were sentenced to pay the victim about 130,000 NOK in compensation (*M* = 134823, SD = 53080).

The dataset was divided into two groups, one including the cases in which there had been a description of IRMAS‐SAFI behavior, *n* = 51, and one group lacking such a description, *n* = 51. Comparisons of the two groups' characteristics are summarized in Table 1. The groups had very similar scores on most of the variables. Only a few cases had any photograph or video evidence available, seven in the IRMAS‐SAFI group and four in the comparison group. Because of the low occurrence, this variable was not included in the chi‐squared test.

**TABLE 1:** Characteristics and comparisons of the groups (*N* = 102).

Variable	IRMAS‐SAFI	Comparison	*t*/*χ* ^*2*^	*p* *
	*M* (SD)/% (*n*)			
Age of defendant	28.93 (6.66)	32.18 (10.09)	1.19	0.059
Year of conviction	2018.59 (2.80)	2017.92 (2.93)	−1.18	0.243
Type of rape				
Incap. rape, not intercourse	13.7% (7)	13.7% (7)	0.00	1.000
Incap. rape, intercourse	43.1% (22)	43.1% (22)		
Force, not intercourse	9.8% (5)	9.8% (5)		
Force, intercourse	33.3% (17)	33.3% (17)		
Degree of violence				
No additional violence	56.9% (29)	56.9% (29)	0.61	0.737
Physical force	33.3% (17)	37.3% (19)		
Excessive violence	9.8% (5)	5.9% (3)		
Place of rape				
Victims home	37.3% (19)	31.4% (16)	1.54	0.672
Defendants home	29.4% (15)	29.4% (15)		
Outside/Public	3.9% (2)	9.8% (5)		
Other	29.4% (15)	29.4% (15)		
Witnesses	6.37 (3.41)	6.47 (2.85)	0.16	0.875
Medical evidence	45.1% (23)	60.8% (31)	2.52	0.113
Logged messages/phone calls	54.9% (28)	43.1% (22)	1.41	0.235
% of female judges (*n* = 93)	0.47 (0.13)	0.44 (0.11)	−1.07	0.287

*Note:* Incap. = incapacitated, IRMAS‐SAFI = Illinois Rape Myth Acceptance Scale—She Asked For It. Incapacitated rape means having sex with someone who is mentally or physically incapacitated, for instance due to alcohol consumption, and are therefore unable to give consent.

* The Bonferroni corrected alpha level is 0.006.

### 2.1 Difference in Sentencing Between the Groups

Defendants in the IRMAS‐SAFI group were given fewer months in prison (*M* = 25.25, SD = 20.90) than defendants in cases where such descriptions did not occur (*M* = 41.65, SD = 13.30). The median values for the two groups were 24 and 48 months. Fourteen of the defendants in the IRMAS‐SAFI group were given no prison sentence (although six of those defendants had to pay a financial penalty), compared to three in the comparison group. No prison sentence given was coded as a score of 0. The difference between the groups was large, Δ*M* = 16.39, *t*(84.78) = 4.73, *p* < 0.001, *d* = 0.94. The result from the Mann–Whitney *U* test was similar, indicating a significant difference in the amount of prison time defendants was given between the groups, *U* = 724.00, *z* = −3.92, *p* < 0.001.

**FIGURE 2:**
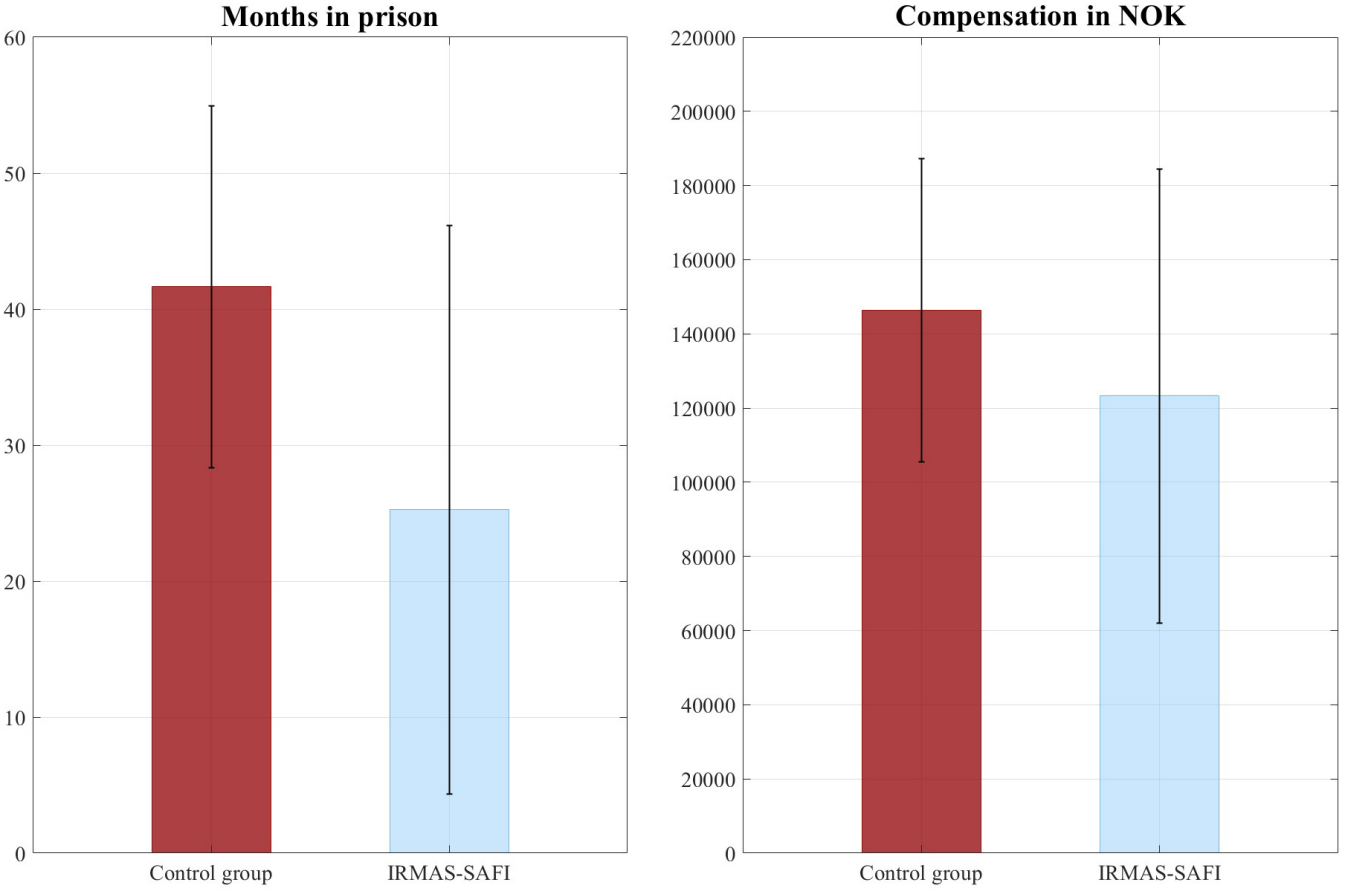
Difference in sentencing between the two groups.

The *t*‐test indicated a small but significant difference in financial penalty for the IRMAS‐SAFI group (*M* = 123255, *SD* = 61185) and the comparison group (*M* = 146392, *SD* = 40925), *t*_(87.28)_ = 2.45, *p* = 0.027, *d* = 0.45. However, the result from the Mann–Whitney *U* test indicated that there was no significant difference between the groups, *U* = 1027.00, *z* = −1.94, *p* = 0.052. The differences in sentences received by defendants in both groups are illustrated in Figure 2.

Because there were more instances of acquittals in the IRMAS‐SAFI group (*n* = 8) than in the comparison group (*n* = 3), a *t*‐test to compare the groups excluding all acquitted cases was also conducted. The difference in prison sentences between the comparison group (*M* = 43. 35, SD = 10.44) and the IRMAS‐SAFI group (*M* = 28.62, SD = 19.95) was still significant, *t*(65.15) = 4.43, *p* < 0.001, and large, *d* = 0.94, after removing acquittals. The median difference between the groups was also significant, *χ* ^2^ = 10.14, *p* = 0.001.

### 2.2 Study Variables Associated With Prison Sentence

Table 1 suggested that there could be a difference between the groups regarding where the rape took place, the age of the defendant, and whether medical evidence was present. Defendants were on average 4 years older in the comparison group than in the IRMAS‐SAFI group. Medical evidence was available in more cases from the comparison group than from the IRMAS‐SAFI group. To investigate whether these variables accounted for any of the difference in the prison sentence variable, a regression analysis was conducted. Whether the rape was violent or forcible was also included as a predictor in the model.

Multicollinearity was not a problem in the regression analysis, as VIF ranged from 1.03 to 1.06, and the Durbin‐Watson test suggested that residuals were not autocorrelated. The largest value for Mahalanobis Distance was 15.96, indicating that there were no problematic outliers. The scatterplot of standardized residuals and predicted values indicated that the assumption of homogeneity of variance was met. Tests of normality and inspection of QQ‐plot suggested that the dependent variable was sufficiently normally distributed.

The model explained a significant proportion of variance in length of prison sentence, *R* ^*2*^ = 0.22, *F*(4, 97) = 7.92, *p* < 0.001. Two of the independent variables (medical evidence and IRMAS‐SAFI) were significantly associated with months in prison. Results from the regression analysis are summarized in Table 2.

**TABLE 2:** Factors associated with months in prison in the original matched samples and robustness tests using a randomly selected control group.

Variable	*B*	SE *B*	*β*	*t*	*p*
Matched comparison group					
Medical evidence (yes/no)	7.20	3.44	0.19	2.09	0.039
Age of defendant	−0.12	0.20	−0.05	−0.60	0.550
Degree of violence (violent/not violent)	6.28	3.42	0.16	1.84	0.069
IRMAS‐SAFI (yes/no)	−15.65	3.48	−0.41	−4.50	< 0.001
Robustness test (randomly selected comparison group)					
Medical evidence (yes/no)	5.19	4.05	0.12	1.28	0.204
Age of defendant	−0.12	0.22	−0.05	−0.54	0.589
Degree of violence (violent/not violent)	19.12	4.11	0.42	4.65	< 0.001
IRMAS‐SAFI (yes/no)	−9.27	4.09	−0.21	−2.27	0.026
All three samples excluding rapes in public places (*n* = 132)					
Medical evidence (yes/no)	7.69	3.15	0.20	2.44	0.016
Age of defendant	−0.01	0.17	−0.01	−0.07	0.948
Degree of violence (violent/not violent)	10.87	3.37	0.26	3.22	0.002
IRMAS‐SAFI (yes/no)	−10.34	3.37	−0.26	−3.07	0.003

*Note:* Violent = rape by force (with and without intercourse), No extra violence = incapacitated rape (with and without intercourse), IRMAS‐SAFI = illinois Rape Myth Acceptance Scale—She Asked For It.

### 2.3 Post Hoc Robustness Test Using a Random Comparison Group

The study results were subjected to a robustness test by including a randomly selected comparison group (Table S3) instead of the original matched comparison group. The new comparison groups received a prison sentence of 33.05 (SD = 22.96) months which was not significantly different from the IRMAS‐SAFI group, *t*(100) = −1.88, *p* = 0.064. However, when controlling for the other variables, the IRMAS‐SAFI variable was significant (*p* = 0.026) in the regression analysis (see Table 2). Degree of violence was the most significant predictor in the regression.

As a final robustness test, the regression was repeated using all three samples but excluding cases where the rape took place at a public place. These were excluded as there was an uneven distribution of such cases in the IRMAS‐SAFI cases (*n* = 2) and the second comparison group (*n* = 14). Presence of medical evidence, degree of violence, and the IRMAS‐SAFI variables were all significant predictors of prison sentence (see Table 2) in the combined sample.

## 3 Discussion

This study examined the effect of descriptions of victims' behavior on sentencing in Norwegian rape trials. The results supported the hypothesis that defendants in cases where the victim is described according to IRMAS‐SAFI receive more lenient sentences. However, there was no clear difference in the amount of compensation the defendants had to pay the victims. This is not surprising, because the evidentiary requirement is more stringent for a prison sentence than for imposing a financial penalty.

The regression analysis indicated that age of the defendant was not significantly associated with length of the prison sentence. However, medical evidence and violent rape were associated with longer prison sentences. When controlling for medical evidence and degree of violence, IRMAS‐SAFI still accounted for a large proportion of variance. This supports the theory that IRMAS‐SAFI descriptions are associated with the severeness of sentencing in rape trials. The findings coincide with research indicating that rape myths affect perception of guilt, blame, and credibility in hypothetical scenarios depicting sexual assault (Ryan and Westera 2018; Berkland, Ji, and Jain 2022). The current study also indicates that this is the case in real rape trials, and that rape myths regarding IRMAS‐SAFI behavior could influence sentencing.

A study on rape myth acceptance among Norwegian lay and professional judges indicated low rates of rape myth acceptance (Bendixen et al. 2014). However, as the study was conducted as a survey the results might be influenced by social desirability bias. It has been suggested that the decrease in acceptance stems from rape myths becoming increasingly unpopular, and acceptance for them thus becoming more subtle (Thelan and Meadows 2022). Also, rape myths may be part of an intuitive, subconscious knowledge, which is seldom reflected on or criticized. Therefore, rape myths could have a larger influence on perception and evaluations than individuals are aware of (Laugerud 2020). As descriptions of victim IRMAS‐SAFI were found to be associated with sentencing in rape trials, the results from this study indicate that rape myth acceptance could possibly influence judges despite previous research indicating low rape myth acceptance (Bendixen et al. 2014).

The results from this study could indicate that IRMAS‐SAFI rape victims are less likely to be believed in court. Consequently, descriptions tapping into rape myths might contribute to unfair sentencing in Norwegian courts. This is problematic, especially considering the proportion of rapes that reach the court is already small. Thus, this study emphasizes the importance of developing measures to prevent rape myths from being presented in court decisions. Increasing the likelihood of conviction in rape cases might be an important step toward raising reporting rates, which in turn could be an important factor in making help more accessible for victims of sexual assault, and to the prevention of future assaults.

A strength of this study is that data were collected from court decisions made by judges in real trials, as opposed to using mock judges and written scenarios. However, a limitation of using real court decisions is that access to information about the complainant, defendant, and judges involved in the rape case is limited. This could mean that the groups were not fully comparable. It is therefore important to consider whether the results could be influenced by other possible third variables. Also, it was not possible to include the judges' level of rape myth acceptance, which could be associated with a higher likelihood of disbelieving rape accusers (Klement, Sagarin, and Skowronski 2019; Leverick 2020; Lilley, Willmott, and Mojtahedi 2023). Several other factors were not included in this study, such as age and ethnicity of the judges, and attractiveness, social status, the relationship between the complainant and defendant, and their ethnicity. Such variables could also influence evaluation of culpability and sentencing in rape cases (Rydberg, Cassidy, and Socia 2018; Cochran et al. 2019; Klement, Sagarin, and Skowronski 2019; Leverick 2020).

Court decisions are made based on the subjective evaluation of judges, based on a set of fixed guidelines as to what is considered aggravating or mitigating. Since rape cases are never identical, and their severity is assessed based on a combination of all the circumstances present, it is impossible to find cases that are completely equivalent. This means that the results obtained in this study may have been partly caused by other factors than judges' perception of the IRMAS‐SAFI descriptions. To combat this effect, the groups were compared on variables regarding severity and evidence base. However, the variables do not perfectly account for actual severity or quality of evidence. For instance, the number of witnesses that testified was extracted from each case, but many witnesses present does not always equal more reliable evidence. This point relates to the issues of applying quantitative methods to social phenomena generally. Trying to quantify complex situations that are evaluated through discretionary overall assessments is problematic, as it simplifies reality.

The IRMAS was not an ideal tool for the purpose of this study, because it is formatted as a questionnaire. This means that the statements are vague, making them less suitable for categorizing cases. Subjective evaluation and interpretation of the different items was therefore necessary, creating a risk of bias. Independent raters were included in the process to reduce this risk. The IRMAS was, however, chosen because previous research using the measure indicates that the myths and ideas the statements convey reflect common and relevant beliefs. This also means that it is easier to relate results to other research on the subject, which often uses the IRMAS.

This study used court decisions from the Norwegian Court of Appeal. There are considerable differences in legal systems, rape legislation, and cultural norms between countries. For instance, rape myth acceptance may be higher in Poland compared to Norway (Łyś et al. 2022). This means that the results may not be readily generalizable outside of Norway, although some research has indicated similar results in rape myth acceptance across different countries (Bendixen, Henriksen, and Nøstdahl 2014). Another limitation of using court decisions is that the texts vary in detail and are not all written in the same way. Additionally, changes in legal procedures such as the discontinuing of the jury in 2018 mean that older court decisions are written differently than more recent court decisions. To reduce the risk of bias caused by these differences, the matching cases for each court decision were as far as it was possible chosen from the same year.

The court decisions used in this study were collected from the Court of Appeal as they had more elaborate descriptions of each case. All these cases have already been tried once, and some of them explicitly state that the verdict was changed because the previous sentence was based on irrelevant information. For instance, in one case the defendant had initially received a shorter sentence because the judges stated that the former sexual relationship between the victim and attacker was to be considered mitigating. It is therefore possible that including cases from the District Court (Tingretten) would reveal an even larger effect. More research is necessary to determine whether this is the case.

There are also important ethical considerations of this study. We only had access to information provided in the published verdicts. Judges may have had access to other relevant information. This risk represents a potential ethical issue related to the interpretation of the findings. Another issue is whether the study could scare victims away from reporting sexual violence due to lack of trust in the criminal justice system and fear of victim blaming. It is important to be aware that IRMAS‐SAFI descriptions were only present in a minority of cases (11.3%). However, increasing awareness that rape myths could influence sentencing in rape trials is also important, because such influence could lead to unfair sentencing. Therefore, including expert testimony might be essential for a well‐informed legal system.

The results from this study indicate that developing and implementing measures to combat the possible influence of rape myths on sentencing in rape trials could be beneficial. One measure that has been proposed is the inclusion of an expert witness testimony in rape trials, to combat stereotypical assumptions about the appropriate or “normal” behavior and characteristics of a legitimate rape victim (Ryan and Westera 2018). Others have suggested providing more information to judges about what types of attitudes contribute to maintaining rape myths (Bendixen et al. 2014) or using less stigmatized labels when discussing cases of sexual violence (Berkland, Ji, and Jain 2022). In conclusion, to make court decisions more just, the development and implementation of measures combating the influence of rape myths in court trials should be prioritized.

## Author Contributions

K.R.R., A.H., and S.S.: conceptualization. K.R.R., T.V., M.G.A., A.H., and S.S.: study design and data collection. K.R.R.: writing – original draft preparation. K.R.R. and S.S.: data processing and analyses. A.H. and S.S.: supervision. K.R.R., T.V., M.G.A., A.H., S.S.: writing – review and editing. All authors contributed to the article and approved the submitted version.

## Ethics Statement

The authors have nothing to report.

## Consent

The authors have nothing to report.

## Conflicts of Interest

The authors declare no conflicts of interest.

## Supporting information


**Table S1.** Dataset containing all 102 court decisions included in the study.

**Table S2.** Text Excerpts from Court Decisions in the Promiscuous Victim Group and their IRMAS coding (*N* = 51).

**Table S3.** Dataset containing 51 court decisions used in the second control group.

## Data Availability

Data are available on request from the authors.
