# Supplementary material for: She Asked for It? Descriptions of Victims' Behaviors Are Associated With Sentencing in Norwegian Rape Trials
Source: Scand J Psychol. 2024 Dec 15;66(3):327–37. doi: 10.1111/sjop.13088 (PMC12042729; doi:10.1111/sjop.13088)
Supplement: Supplementary file 1 — Table S1. Dataset containing all 102 court decisions included in the study. [file SJOP-66-327-s003.docx]

| **Table S1** |  |  |  |  |  |  |  |  |  |  |  |  |  |  |
| --- | --- | --- | --- | --- | --- | --- | --- | --- | --- | --- | --- | --- | --- | --- |
| *Dataset containing all 102 court decisions included in the study* | | | | | | | | | | | | | | |
| **Court decision** | **IRMAS-SAFI^a^** | **Type of rape ^b^** | **Age Defendant** | **Penalty Prison** | **Penalty NOK** | **Year Conviction** | **Witnesses** | **Med Evidence ^a^** | **SMSor PhCa ^a^** | **Degree Violence ^c^** | **Place of rape ^d^** | **Photo or Video ^a^** | **Female Judges (%)** | **Nr. Items IRMAS** |
| LE-2013-124825 | 0 | 4 | 61 | 48 | 150000 | 2014 | 7 | 1 | 1 | 2 | 1 | 0 | 43 | 0 |
| LF-2017-17537 | 0 | 1 | 48 | 39 | 125000 | 2017 | 8 | 1 | 0 | 1 | 1 | 0 | 29 | 0 |
| LH-2013-81353 | 0 | 1 | 52 | 39 | 120000 | 2013 | 10 | 0 | 0 | 1 | 1 | 0 | 57 | 0 |
| LH-2017-192913 | 0 | 4 | 27 | 0 | 150000 | 2018 | 9 | 0 | 1 | 2 | 3 | 0 |  | 0 |
| LG-2016-3002 | 0 | 4 | 36 | 48 | 150000 | 2016 | 10 | 0 | 0 | 2 | 1 | 0 | 29 | 0 |
| LB-2013-179955 | 0 | 2 | 30 | 42 | 150000 | 2014 | 5 | 1 | 1 | 1 | 1 | 0 | 43 | 0 |
| LB-2016-29878 | 0 | 2 | 31 | 50 | 175000 | 2016 | 7 | 0 | 0 | 1 | 1 | 0 | 43 | 0 |
| LF-2014-61995 | 0 | 2 | 28 | 48 | 150000 | 2014 | 7 | 1 | 0 | 1 | 1 | 0 | 43 | 0 |
| LG-2015-78777 | 0 | 2 | 28 | 48 | 175000 | 2016 | 4 | 1 | 0 | 1 | 1 | 0 | 43 | 0 |
| LG-2017-108844 | 0 | 4 | 29 | 54 | 150000 | 2018 | 9 | 0 | 0 | 2 | 1 | 0 | 43 | 0 |
| LB-2023-8638 | 0 | 2 | 29 | 0 | 0 | 2023 | 13 | 1 | 1 | 1 | 1 | 0 | 29 | 0 |
| LH-2022-101606 | 0 | 2 | 27 | 48 | 223000 | 2022 | 4 | 1 | 1 | 1 | 1 | 0 | 43 | 0 |
| LG-2022-164654 | 0 | 2 | 26 | 42 | 220000 | 2023 | 7 | 1 | 0 | 1 | 1 | 0 | 43 | 0 |
| LG-2020-97402 | 0 | 4 | 21 | 48 | 150000 | 2021 | 3 | 0 | 1 | 3 | 4 | 0 | 57 | 0 |
| LG-2023-107880 | 0 | 2 | 24 | 48 | 240000 | 2023 | 6 | 1 | 0 | 1 | 1 | 1 | 57 | 0 |
| LB-2015-14776 | 0 | 4 | 33 | 48 | 150000 | 2015 | 6 | 0 | 0 | 2 | 4 | 0 | 57 | 0 |
| LE-2017-40849 | 0 | 4 | 35 | 66 | 150000 | 2017 | 4 | 1 | 0 | 2 | 2 | 0 | 57 | 0 |
| LA-2019-139919 | 0 | 4 | 23 | 45 | 150000 | 2020 | 5 | 1 | 0 | 2 | 1 | 0 | 43 | 0 |
| LG-2016-151663 | 0 | 4 | 31 | 48 | 150000 | 2017 | 13 | 1 | 0 | 2 | 2 | 0 | 43 | 0 |
| LA-2015-69162 | 0 | 3 | 24 | 50 | 125000 | 2015 | 0 | 1 | 0 | 2 | 2 | 0 | 43 | 0 |
| LA-2022-58120 | 0 | 3 | 30 | 4 | 70000 | 2022 | 6 | 0 | 1 | 2 | 2 | 0 | 43 | 0 |
| LA-2016-165851 | 0 | 3 | 21 | 39 | 130000 | 2017 | 10 | 1 | 0 | 2 | 2 | 0 | 43 | 0 |
| LH-2018-163419 | 0 | 2 | 62 | 48 | 150000 | 2019 | 4 | 1 | 1 | 1 | 3 | 0 | 43 | 0 |
| LG-2018-146813 | 0 | 1 | 51 | 38 | 150000 | 2019 | 5 | 0 | 0 | 1 | 3 | 0 | 43 | 0 |
| LB-2013-7178 | 0 | 2 | 33 | 45 | 150000 | 2013 | 4 | 1 | 0 | 1 | 4 | 0 | 43 | 0 |
| LG-2018-1785 | 0 | 4 | 43 | 48 | 150000 | 2018 | 7 | 1 | 1 | 2 | 3 | 0 | 29 | 0 |
| LB-2012-177442 | 0 | 2 | 32 | 48 | 150000 | 2013 | 7 | 0 | 1 | 1 | 3 | 0 | 57 | 0 |
| LF-2015-203629 | 0 | 2 | 34 | 48 | 150000 | 2016 | 2 | 1 | 1 | 1 | 3 | 0 | 29 | 0 |
| LG-2015-88601 | 0 | 4 | 32 | 48 | 150000 | 2016 | 7 | 0 | 0 | 2 | 3 | 0 | 29 | 0 |
| LB-2021-15593 | 0 | 1 | 34 | 39 | 125000 | 2021 | 1 | 0 | 0 | 1 | 1 | 0 | 43 | 0 |
| LB-2016-30420 | 0 | 3 | 28 | 34 | 125000 | 2016 | 11 | 1 | 0 | 2 | 3 | 0 |  | 0 |
| LB-2018-145485 | 0 | 2 | 30 | 48 | 175000 | 2018 | 6 | 1 | 0 | 1 | 3 | 1 | 43 | 0 |
| LH-2019-159020 | 0 | 4 | 29 | 40 | 150000 | 2020 | 7 | 1 | 0 | 2 | 3 | 0 | 57 | 0 |
| LB-2021-147151 | 0 | 4 | 30 | 0 | 0 | 2022 | 12 | 0 | 1 | 3 | 3 | 0 | 57 | 0 |
| LA-2018-94371 | 0 | 4 | 25 | 52 | 175000 | 2017 | 4 | 1 | 0 | 2 | 3 | 0 | 57 | 0 |
| LB-2018-125155 | 0 | 2 | 24 | 48 | 150000 | 2019 | 5 | 1 | 0 | 1 | 3 | 0 | 29 | 0 |
| LB-2018-106436 | 0 | 1 | 50 | 36 | 150000 | 2018 | 5 | 1 | 1 | 1 | 1 | 0 | 57 | 0 |
| LG-2018-168849 | 0 | 2 | 22 | 48 | 150000 | 2019 | 10 | 1 | 1 | 1 | 3 | 0 | 43 | 0 |
| LB-2013-38270 | 0 | 3 | 42 | 30 | 125000 | 2013 | 8 | 1 | 1 | 2 | 3 | 0 | 57 | 0 |
| LB-2015-54810 | 0 | 2 | 42 | 42 | 150000 | 2015 | 4 | 1 | 0 | 1 | 4 | 0 | 43 | 0 |
| LB-2017-155827 | 0 | 4 | 41 | 42 | 150000 | 2018 | 8 | 0 | 0 | 2 | 4 | 0 | 29 | 0 |
| LB-2018-25829 | 0 | 2 | 34 | 46 | 150000 | 2018 | 4 | 0 | 1 | 1 | 4 | 0 | 43 | 0 |
| LB-2016-176841 | 0 | 2 | 30 | 48 | 150000 | 2017 | 6 | 0 | 1 | 1 | 4 | 0 | 29 | 0 |
| LH-2019-155441 | 0 | 2 | 33 | 48 | 150000 | 2020 | 4 | 1 | 0 | 1 | 4 | 0 | 29 | 0 |
| LB-2016-60352 | 0 | 2 | 25 | 48 | 150000 | 2016 | 6 | 0 | 0 | 1 | 4 | 0 | 43 | 0 |
| LB-2015-153302 | 0 | 2 | 24 | 43 | 175000 | 2016 | 6 | 1 | 1 | 1 | 4 | 0 | 43 | 0 |
| LB-2021-103719 | 0 | 1 | 27 | 34 | 120000 | 2022 | 6 | 0 | 1 | 1 | 4 | 1 | 57 | 0 |
| LG-2019-102435 | 0 | 4 | 23 | 48 | 150000 | 2020 | 9 | 1 | 1 | 3 | 4 | 0 | 57 | 0 |
| LF-2021-114408 | 0 | 1 | 21 | 39 | 120000 | 2021 | 4 | 0 | 0 | 1 | 4 | 1 | 43 | 0 |
| LA-2022-164777 | 0 | 2 | 20 | 44 | 223000 | 2023 | 10 | 1 | 1 | 1 | 4 | 0 | 71 | 0 |
| LH-2020-105516 | 0 | 4 | 21 | 42 | 150000 | 2020 | 5 | 0 | 1 | 2 | 4 | 0 | 57 | 0 |
| LA-2020-106596 | 1 | 4 | 26 | 16 | 90000 | 2020 | 8 | 1 | 0 | 2 | 4 | 0 | 57 | 3 |
| LG-2015-121228 | 1 | 4 | 47 | 9 | 150000 | 2016 | 7 | 0 | 0 | 2 | 1 | 0 | 29 | 2 |
| LB-2021-182486 | 1 | 2 | 40 | 40 | 220000 | 2022 | 14 | 1 | 1 | 1 | 1 | 0 | 57 | 3 |
| LB-2015-54804 | 1 | 2 | 31 | 0 | 0 | 2015 | 3 | 1 | 1 | 1 | 1 | 0 |  | 1 |
| LH-2018-152038 | 1 | 2 | 34 | 0 | 0 | 2018 | 6 | 0 | 1 | 1 | 1 | 1 | 43 | 2 |
| LB-2019-159726 | 1 | 3 | 34 | 0 | 0 | 2020 | 7 | 0 | 1 | 2 | 1 | 1 | 71 | 2 |
| LB-2017-139018 | 1 | 4 | 30 | 48 | 150000 | 2018 | 3 | 1 | 1 | 3 | 1 | 1 | 43 | 2 |
| LG-2018-180512 | 1 | 4 | 31 | 48 | 150000 | 2019 | 6 | 1 | 0 | 2 | 1 | 0 | 43 | 2 |
| LG-2022-97372 | 1 | 2 | 34 | 36 | 220000 | 2022 | 6 | 0 | 0 | 1 | 1 | 0 | 29 | 2 |
| LH-2017-50045 | 1 | 2 | 29 | 44 | 150000 | 2017 | 4 | 0 | 1 | 1 | 1 | 0 | 57 | 1 |
| LA-2013-22340 | 1 | 2 | 24 | 20 | 90000 | 2013 | 8 | 1 | 0 | 1 | 1 | 0 | 43 | 1 |
| LB-2022-112954 | 1 | 4 | 32 | 48 | 220000 | 2022 | 8 | 1 | 1 | 2 | 1 | 0 | 29 | 2 |
| LB-2018-119700 | 1 | 2 | 28 | 12 | 90000 | 2019 | 3 | 1 | 1 | 1 | 1 | 0 | 57 | 3 |
| LF-2020-47258 | 1 | 2 | 29 | 48 | 150000 | 2020 | 8 | 1 | 1 | 1 | 1 | 0 |  | 1 |
| LG-2020-182646 | 1 | 2 | 29 | 0 | 150000 | 2021 | 10 | 0 | 1 | 1 | 1 | 0 | 29 | 1 |
| LH-2016-152840 | 1 | 4 | 23 | 42 | 150000 | 2017 | 6 | 0 | 0 | 2 | 1 | 0 | 43 | 4 |
| LH-2018-181738 | 1 | 4 | 25 | 50 | 170000 | 2019 | 8 | 1 | 1 | 2 | 1 | 0 | 43 | 1 |
| LB-2017-168865 | 1 | 2 | 22 | 0 | 150000 | 2019 | 3 | 0 | 1 | 1 | 1 | 0 | 71 | 3 |
| LF-2020-52355 | 1 | 2 | 27 | 18 | 90000 | 2020 | 7 | 0 | 1 | 1 | 1 | 0 | 43 | 1 |
| LB-2022-24515 | 1 | 1 | 24 | 19 | 65000 | 2022 | 5 | 0 | 0 | 1 | 1 | 0 | 57 | 2 |
| LE-2023-472 | 1 | 3 | 36 | 4 | 80000 | 2023 | 3 | 1 | 1 | 2 | 2 | 0 | 57 | 2 |
| LH-2015-179477 | 1 | 3 | 25 | 30 | 110000 | 2016 | 8 | 0 | 0 | 2 | 2 | 0 | 29 | 1 |
| LA-2015-176696 | 1 | 2 | 34 | 0 | 150000 | 2016 | 8 | 0 | 1 | 1 | 3 | 0 |  | 1 |
| LA-2013-109478 | 1 | 4 | 31 | 48 | 150000 | 2014 | 2 | 1 | 0 | 3 | 3 | 1 | 43 | 2 |
| LF-2014-205143 | 1 | 3 | 30 | 40 | 150000 | 2015 | 12 | 0 | 0 | 3 | 3 | 0 | 43 | 3 |
| LA-2021-162674 | 1 | 4 | 37 | 48 | 150000 | 2022 | 5 | 1 | 1 | 2 | 3 | 0 | 29 | 2 |
| LA-2018-142393 | 1 | 4 | 33 | 0 | 175000 | 2019 | 15 | 1 | 0 | 2 | 3 | 0 | 43 | 2 |
| LG-2021-81411 | 1 | 4 | 34 | 0 | 150000 | 2021 | 3 | 1 | 1 | 2 | 3 | 0 | 43 | 2 |
| LG-2017-87191 | 1 | 4 | 25 | 69 | 150000 | 2017 | 2 | 0 | 1 | 3 | 3 | 0 | 43 | 2 |
| LB-2015-183117 | 1 | 2 | 22 | 0 | 90000 | 2016 | 6 | 0 | 0 | 1 | 3 | 0 |  | 3 |
| LB-2016-190401 | 1 | 4 | 23 | 48 | 150000 | 2017 | 3 | 0 | 0 | 2 | 4 | 0 | 43 | 1 |
| LH-2015-10849 | 1 | 4 | 20 | 14 | 90000 | 2015 | 4 | 0 | 1 | 2 | 3 | 0 | 57 | 3 |
| LA-2016-205121 | 1 | 4 | 21 | 54 | 175000 | 2017 | 13 | 1 | 1 | 2 | 3 | 0 | 43 | 2 |
| LH-2018-187180 | 1 | 2 | 23 | 48 | 150000 | 2019 | 12 | 1 | 1 | 1 | 3 | 0 | 57 | 2 |
| LG-2023-91519 | 1 | 2 | 26 | 0 | 0 | 2023 | 6 | 0 | 0 | 1 | 3 | 0 | 43 | 2 |
| LH-2021-2988 | 1 | 2 | 24 | 44 | 150000 | 2021 | 7 | 1 | 1 | 1 | 3 | 0 | 57 | 1 |
| LG-2022-67463 | 1 | 4 | 22 | 48 | 223000 | 2022 | 3 | 0 | 1 | 2 | 3 | 1 | 43 | 2 |
| LH-2014-207080 | 1 | 2 | 46 | 12 | 90000 | 2015 | 1 | 0 | 0 | 1 | 4 | 0 |  | 2 |
| LB-2016-203813 | 1 | 3 | 42 | 42 | 160000 | 2017 | 3 | 1 | 0 | 3 | 4 | 0 | 43 | 2 |
| LB-2017-124959 | 1 | 4 | 41 | 45 | 150000 | 2018 | 9 | 0 | 0 | 2 | 4 | 0 | 43 | 1 |
| LG-2018-190076 | 1 | 2 | 34 | 0 | 0 | 2019 | 3 | 0 | 0 | 1 | 4 | 0 | 86 | 3 |
| LF-2017-174694 | 1 | 2 | 32 | 0 | 0 | 2018 | 10 | 1 | 1 | 1 | 4 | 0 |  | 1 |
| LH-2018-152693 | 1 | 2 | 29 | 0 | 100000 | 2019 | 7 | 1 | 1 | 1 | 4 | 0 | 57 | 2 |
| LH-2021-99640 | 1 | 1 | 29 | 42 | 150000 | 2021 | 6 | 0 | 0 | 1 | 4 | 1 | 71 | 2 |
| LG-2013-105162 | 1 | 1 | 20 | 6 | 80000 | 2013 | 7 | 0 | 0 | 1 | 4 | 0 | 43 | 2 |
| LH-2015-129838 | 1 | 2 | 23 | 0 | 90000 | 2016 | 14 | 0 | 1 | 1 | 4 | 0 | 43 | 1 |
| LG-2013-201799 | 1 | 2 | 21 | 12 | 70000 | 2014 | 1 | 0 | 0 | 1 | 4 | 0 |  | 4 |
| LF-2023-147742 | 1 | 1 | 26 | 24 | 240000 | 2023 | 5 | 0 | 0 | 1 | 4 | 0 | 43 | 1 |
| LG-2020-160556 | 1 | 1 | 24 | 39 | 120000 | 2021 | 6 | 0 | 1 | 1 | 4 | 0 | 57 | 1 |
| LB-2023-40578 | 1 | 1 | 22 | 34 | 165000 | 2023 | 6 | 1 | 1 | 1 | 4 | 0 | 43 | 2 |
| LH-2019-119406 | 1 | 1 | 22 | 39 | 120000 | 2019 | 5 | 1 | 0 | 1 | 3 | 0 | 29 | 2 |
| *Note. ^a^ 1 = Yes, 0 = No. ^b^ 1 = Incapacitated rape, not intercourse, 2 = Incapacitated rape, intercourse, 3 = Rape by force, not intercourse, 4 = Rape by force, intercourse. ^c^ 1 = No additional violence, 2 = Physical force, 3 = Excessive violence. ^d^ 1 = Home, 2 = Outside or in public, 3 = Defendants’ home, 4 = Other.* | | | | | | | | | | | | | | |
