# Supplementary material for: She Asked for It? Descriptions of Victims' Behaviors Are Associated With Sentencing in Norwegian Rape Trials
Source: Scand J Psychol. 2024 Dec 15;66(3):327–37. doi: 10.1111/sjop.13088 (PMC12042729; doi:10.1111/sjop.13088)
Supplement: Supplementary file 2 — Table S2. Text Excerpts from Court Decisions in the Promiscuous Victim Group and their IRMAS coding (N = 51). [file SJOP-66-327-s001.docx]

**Table S2**

*Text Excerpts from Court Decisions in the Promiscuous Victim Group and their IRMAS coding (N = 51)*

| **Code** | **IRMAS- item** | **Interpretation** |  |  |
| --- | --- | --- | --- | --- |
| **1** | “When girls go to parties wearing slutty clothes, they are asking for trouble” | All remarks of the woman wearing little clothing, e.g. short skirts and dresses. |  |  |
| **2** | “If a girl goes to a room alone with a guy at a party, it is her own fault if she is raped” | All instances of the woman going home with the defendant or joining him in a room or bed alone at a party (except when this is done for a stated purpose such as fetching something or vomiting). |  |  |
| **3** | “If a girl acts like a slut, eventually she is going to get into trouble” | All descriptions of the woman having participated in casual sexual/intimate activities with one or several people other than the defendant, e.g. cheating on partner or making out with others at a party. |  |  |
| **4** | “When girls are raped, it’s often because the way they said “no” was unclear” | All remarks of the woman not expressing her unwillingness to have sex clearly enough. |  |  |
| **5** | “If a girl initiates kissing or hooking up, she should not be surprised if a guy assumes she wants to have sex” | All instances of the complainant having engaged voluntarily in intimate activities (e.g. kissing, cuddling, sex) with the defendant prior to the assault. |  |  |
| **Court decision** | **Text excerpts (translated from Norwegian)** | | **Coded as** |  |
| **LB-2016-203813** | “The fact that the defendant and the *complainant agreed to have sexual intercourse* earlier in the evening cannot have any bearing on the sentencing. It is also completely irrelevant what *voluntary sexual activity may have taken place between the parties* until the defendant became brutal and the complainant asked him to stop.” | | 2, 5 |  |
| **LH-2021-99640** | “The complainant was extremely intoxicated at the party, *she kissed several people* and had part of her zipper fly open. This shows a high level of sexual tension and a desire for sexual activity.”  “In addition, the fact that *she voluntarily had sex with two men* during the course of [date], does not appear to be compatible with her claim that she was assaulted on the night of [date].” | | 1, 3 |  |
| **LG-2018-190076** | “In fact, the Court of Appeal finds it proven that the defendant and the complainant know each other from growing up at [location], that they are friends, *flirt at parties and that they have had intimate contact of a sexual nature on several occasions*.”  “E further explained that *the defendant kissed the complainant on the neck and throat, and that they became very close and intimate*.”  “The witness felt *they were too close due to the fact that the complainant had a boyfriend.*”  “According to the sister, *only the complainant and the defendant were left at the table when she went to bed*.” | | 2,3,5 |  |
| **LG-2021-81411** | “When *they arrived at the defendant's apartment,* the defendant went to the bathroom. The complainant, who then went to the bathroom, took off her clothes and got into bed. The defendant then came out of the bathroom, undressed and got into bed. *They started kissing and touching each other.*”  “The complainant then turned around at some point, and the purpose was undoubtedly that they should continue to have voluntary intercourse. The complainant wanted them to continue with vaginal sex.” | | 2, 5 |  |
| **LB-2022-112954** | “A and B made contact during the evening. *They talked a lot, danced and kissed.* Between [time] and [time], they took a taxi together to B's flat. She was the one who paid for the taxi, and this happened at [time] according to the receipt. *B took the initiative for them to go to her home.*”  “It is agreed that this part of the sexual intercourse was voluntary, and it is not necessary for the Court of Appeal to take a closer look at the differences in the statements.” | | 2, 5 |  |
| **LB-2017-168865** | “*The complainant and C already had a sexual relationship but were not in a committed relationship with each other*.”  “She had on a previous occasion had sex with both C and another man in her home, and wonders whether C may have mentioned this to the accused.” | | 3 |  |
| **LG-2023-91519** | “There, *the defendant and the complainant had sat together and kissed.* The defendant ordered a taxi and the complainant and D got in. *The two ended up going to the defendant's home to sleep there*.”  “The defendant's bed was a double bed. *He and the complainant laid under the same duvet*. He laid on his back in the middle of the bed, but she laid on his left side, facing the defendant and with her hand on his chest. While lying like this, *they kissed and fell asleep in this position.*” | | 2, 5 |  |
| **LB-2021-182486** | “Throughout the fall of [year], the defendant and the complainant had contact with each other outside of working hours. The Court of Appeal assumes that they entered into a *relationship of friendship, which also included physical advances*.”  “The weekend before the incident in the indictment, the defendant and the complainant went on a trip to [location]. The defendant's partner was not aware of this.” “When they arrived in [location], they checked into a double room where they both stayed for two nights. It is undisputed that the defendant and *the complainant had sex* during their stay in [location].”  “*The sexual activity was voluntary on her part*. At the same time, she felt somewhat pressured in the situation and ambivalent about *having sex with the defendant, who was her boss and whom she knew had a partner and small children*.”  “A photo of the complainant and E on the evening in question has been submitted, showing that *the complainant was wearing a short black dress* and high boots.” | | 1, 3, 5 |  |
| **LG-2020-182646** | “In brief, the background to the case is that the defendant and the complainant met each other at the [name] nightclub in central [location] on [date] at around [time]. They had both been drinking alcohol, and at around [time] *it was decided that they would take a taxi together*.”  “*The taxi ride that ended at the complainant's home* was paid for at [time].” | | 2 |  |
| **LB-2023-40578** | “B explained that she, D, A and C drank alcohol and snorted cocaine. During the evening, *she and C had sex*. This was at the request of both of them.”  “She then realized that it was another person who was fingering her. The person was behind her. B said she became cold, she shivered, and *she didn't dare to move*." | | 3, 4 |  |
| **LH-2018-187180** | “They agreed that *the complainant should come to the defendant's apartment*, and both have explained that *they kissed inside the apartment*.” | | 2, 5 |  |
| **LA-2016-205121** | “They belonged to the same group of friends, and they had also *had sexual contact on several occasions*.”  “She therefore sent him a text message at [time] on the night of [date], in which *she asked to sleep with him* and said that they had something to talk about.”  “The victim has explained that she noticed that the defendant seemed determined or a little angry when he arrived. They nevertheless *gave each other a kiss and went into the house*.” | | 2, 5 |  |
| **LB-2017-139018** | “B has further explained that it was “in the cards” that *they would have sexual intercourse at her home, and that she accepted this*. When they arrived, they undressed quite quickly.” | | 2, 5 |  |
| **LG-2013-105162** | “At the party, it is not disputed that the defendant and the complainant hung out together, that *she sat on his lap and that there was some kissing*.”  “The defendant and his friend, D, followed the two women back to the hotel, and all four of them *ended up spending the night in the same bed.*” | | 2, 5 |  |
| **LB-2016-190401** | “A arrived at the room some time after [time] after B had sent him an SMS with the room number and the code for the front door. Based on the evidence, the Court of Appeal assumes that *A and B first laid in the same bed in the hotel room* and watched a movie.” | | 2 |  |
| **LB-2015-183117** | “B and A were a couple for a few months in [date], and they resumed contact in [date]. On the evening in question in [year], they went to a pre-party together and then to a bowling alley, before *ending up in A's apartment* after midnight.”  “*Nor is it stated that at any time during the intercourse, B told A that she did not want to have intercourse*.” | | 2, 4, 5 |  |
| **LB-2015-54804** | “In brief, the background to the case is that *B invited A to her home* on the night of [date], after she had had a pre-party with some friends who then went out on the town without her wanting to join them.” | | 2 |  |
| **LG-2017-87191** | “The complainant, who *was a prostitute* and 18 years old at the time, offered her services online.”  “*The complainant followed the defendant into the apartment* at [address] at approximately [time].” | | 2, 3 |  |
| **LF-2014-205143** | “*The defendant and the complainant eventually developed a sexual relationship while the complainant and C were still a couple*.”  “She drove to the defendant's house after the movie and *ended up spending the night there*. At this time, the defendant lived on a farm at [location].” | | 2, 3, 5 |  |
| **LH-2018-181738** | “*The defendant and the complainant had a sexual relationship* in the period [date] to the fall of [year]. Since then, they have had sporadic contact on social media. *This contact was occasionally also of a sexual nature*.” | | 5 |  |
| **LA-2013-109478** | “It is undisputed that A and B met at the [name] nightclub in [location] on the night of [date]. At closing time, *they agreed to continue the party in his apartment* and took a taxi there together.”  “The taxi driver's statement confirms that *they started kissing and making* out already in the taxi, and that the atmosphere was good. She said she didn't want to have sex with him, which he said was fine. However, the driver did not perceive this as being entirely serious, but rather as a form of teasing.” | | 2, 5 |  |
| **LB-2017-124959** | “When they stood up, he held her hand and they went to the elevator. There he pressed her against the wall and *they kissed*. Her sister came by, and when asked, B replied that “everything was fine”. *They kissed some more*, but she said she wanted to go back to her friends.” | | 5 |  |
| **LG-2015-121228** | "Based on the testimony, the Court of Appeal assumes that the relationship lasted until the beginning of [year], after which the parties broke up. They nevertheless had considerable contact, particularly with the exchange of a very large number of Facebook messages. *They also had some sexual contact*, most recently at [date].”  “*The defendant was invited in*. They agreed to talk together." | | 2, 5 |  |
| **LA-2015-176696** | “They stayed in the defendant's apartment throughout the evening and night and played games, watched TV and drank alcohol. During the evening, *the complainant voluntarily had sexual intercourse with E.*” | | 3 |  |
| **LG-2018-180512** | “At [location], the defendant and the complainant talked together, and they eventually stood outside the building and *kissed each other*. According to the complainant, *the defendant had a hand under her dress*, and the defendant has not ruled this out.”  “She has explained that she did not like the fact that he did it in public, but that *she did not mind*.”  “*The defendant and the complainant took a taxi together to the complainant's home* at the time.” | | 2, 5 |  |
| **LA-2013-22340** | “The Court of Appeal also assumes that *she flirted and “made out” with the defendant* while they were at [location].” | | 5 |  |
| **LF-2017-174694** | “D was sitting in the front passenger seat of the taxi. He observed that *A and B, who were sitting in the back seat, were kissing/fondling each other* during the journey. B was leaning over A while this was going on.” | | 5 |  |
| **LA-2018-142393** | “After a few years as friends, they became lovers and cohabitants in [year]. It is undisputed that their sexual life was important to both the defendant and the complainant, *that they had frequent sex with each other, and experimented with various forms of sex*. They also included third parties in their shared sexual life.”  “In [year], the defendant and the complainant had their first son, C. They married in [year]. The same fall, *the complainant had a sexual relationship with another man*.” | | 5, 3 |  |
| **LH-2018-152693** | “She and C went into the bathroom and bathed together in the bathtub.”  “While the defendant was in the bathroom, *she was wearing a bra and panties*.”  “There was no sexual contact between the defendant and the complainant. After the defendant and C's brother had left the bathroom, *the complainant and C had sex*.” | | 1, 3 |  |
| **LG-2022-97372** | “When they were at the after-party, they went outside and had a smoke, and *they started kissing*. *The defendant and B agreed to go to her home*, at [address].”  “Both B and the defendant have explained that *they had voluntary vaginal sex* in the bedroom at [address], as well as *performing oral sex on each other*.” | | 2, 5 |  |
| **LA-2021-162674** | “During the evening, *the complainant and the defendant flirted with each other*, and when the nightclubs closed for the night, *the two went home to the defendant*. They both say that they had an expectation that they would have sex with each other.”  “In any case, *they “made out” with each other* and partially undressed before going up to the bedroom upstairs.” | | 2, 5 |  |
| **LG-2022-67463** | “It is undisputed that *voluntary sexual intercourse* took place between the defendant and the complainant when *they arrived at the defendant's home* at [location].” | | 2, 5 |  |
| **LH-2016-152840** | “The defendant and the complainant had established contact on the website Tinder about three months before they met in [location] on the weekend of [date]. Both have explained that *much of the online contact was sexually related*, and that they talked a lot about sex before they met. However, they did not have a romantic relationship. The evidence shows that *the complainant had a sexual relationship with another man in the fall of* [year].”  “In any case, it is clear that *the defendant and the complainant met in the bathroom* during the pre-party, where *the complainant performed oral sex on the defendant* by sucking his penis. The complainant has explained that this was voluntary on her part.”  “The court assumes that *the complainant went into the bedroom and laid down with the defendant* and that *she took off all her clothes except her panties*.” | | 1, 2, 3, 5 |  |
| **LH-2015-129838** | “A unanimous Court of Appeal assumes *that A and B flirted with each other at the after-party, including by kissing each other.* This is based on the testimony of witness C, who observed that they stood at the kitchen counter and kissed and caressed each other.” | | 5 |  |
| **LH-2015-179477** | “It is undisputed that they have known each other for a long time and have also been very good friends with *sporadic sexual relations* in [year] and on one occasion in spring [year].” | | 5 |  |
| **LE-2023-472** | “On the dance floor, they danced closely with *a lot of body contact and touching*. Both the defendant and the complainant participated actively in this.”  “*There was mutual physical contact*.”  “Shortly thereafter, the defendant quickly put his left hand under *the complainant's short skirt*, pulled her panties to the side and inserted one or more fingers into her vagina from behind.” | | 1, 5 |  |
| **LH-2014-207080** | “However, she remained sitting next to C, with whom *she was flirting and making out*, and they sat holding each other until they both fell asleep.”  “It is unclear to the court whether *the complainant had taken off her trousers* at this time or whether it was the defendant who did this.”  “The court furthermore finds it undoubted that *the victim lay with her back against his body and pressed her butt against his abdomen.*” | | 1, 3 |  |
| **LH-2015-10849** | “After a while, *they went to the defendant's bedsit*. C ended up sleeping in the defendant's bed, while *the defendant and the complainant slept on a single mattress on the floor*. The complainant fell asleep quickly, and she has explained that *the defendant may have fondled her arms, back, stomach and thighs*.”  “They woke up early [time]. They talked and flirted, and there was a flirtatious tone. *The complainant explained that she thinks C unhooked her bra at the back and touched her breasts a little*.”  “She and A talked about sex. *He was allowed to touch her breasts*. She said *she liked to be fondled between her thighs, and he did*.” | | 2, 3, 5 |  |
| **LG-2013-201799** | “The two girls are said to have *removed their tops so that they were sitting in bras. The light was turned off and they “touched each other on the buttocks, breasts and in the crotch, outside the clothes.”*  “After the complainant, C and the defendant had laid down in the double bed, the complainant and C soon started having sex while the defendant sat and watched. The complainant then allegedly stated that it was not “fair” to the defendant that he just sat and watched, and after some discussion in which the relationship between the defendant and D is said to have been one of the topics, *the three of them ended up having sex together.*”  “They then went to sleep. *The defendant laid at the far end of the wall, the complainant in the middle, and C at the far end. They laid in a “spoon” position*, i.e. they lay with their backs to each other.” | | 1, 2, 3, 5 |  |
| **LH-2021-2988** | “In the morning, B went there. She did not speak to the defendant. *She and C went into a bedroom and kissed and made out*. A eventually knocked on the door, and both C and B went out into the hallway.” | | 3 |  |
| **LB-2018-119700** | “*B and A met and had sexual intercourse* on a number of occasions over a few months in the fall of [year], the last time on [date]. After this, they did not meet again until [date].”  *“Both A and B have explained that they laid in bed together under one duvet. They “spooned”*, and A held B. *B was wearing a thong, but was otherwise naked.*”  “In the majority's view, *there is nevertheless reason to judge the present case somewhat less strictly*. In contrast to the decision from [year], the defendant did not enter the apartment or the complainant's bed uninvited, but *was invited by the complainant*, first to a party and eventually also *to her apartment* after they had been at a nightclub. Moreover, *the complainant did not object to them spooning. She was wearing a thong, but was otherwise naked. The defendant and the complainant knew each other well sexually and had had sex for several months.*” | | 1, 2, 5 |  |
| **LF-2023-147742** | “Both have explained that they had some contact along the way. *They kissed each other*, but explained that they were just friends and that this did not lead to anything more.” | | 5 |  |
| **LB-2022-24515** | “B and A hit it off, and *they flirted and kissed.* After a while, the five of them walked together to [location]. Later, they took the bus to B's apartment in [location]. On the bus, *B and A sat next to each other and held hands*.”  “*B and A laid down in her bed.*” | | 2, 5 |  |
| **LH-2018-152038** | “On [date], the defendant came to [location]. He had a [nationality] friend (C) with him. Both of them were to stay with B in her home. *The defendant was to sleep in B's bedroom*.”  “During the main hearing, B explained that *the defendant had had sexual intercourse with her* on [date] at her home.” | | 2, 5 |  |
| **LH-2017-50045** | “During the course of the evening, the complainant and the defendant danced with each other next to a table at the bar, and on one occasion *they gave each other a brief kiss*.” | | 5 |  |
| **LF-2020-52355** | “They gradually developed a closer relationship, which also *involved sexual contact, but they did not consider themselves to be a couple*.” | | 5 |  |
| **LB-2019-159726** | "The meeting made them both want to get to know each other better. They met the next morning for a run. They agree *that there was a kiss when they parted ways* but disagree on who kissed whom.”  “When they entered the apartment, the defendant laid down on the sofa. *The complainant laid down next to the defendant, and he began to stroke her stomach outside her clothes and kissed her several times*.” | | 2, 5 |  |
| **LF-2020-47258** | “On the stairs, *B and F made out with each other, and they repeated this on the sofa* in the attic.” | | 3 |  |
| **LG-2020-160556** | "They developed a friendship. *They had sexual intercourse* a couple of times, *but they were not a couple*.” | | 5 |  |
| **LA-2020-106596** | “Both have explained that *they kissed and made out* with each other on the way to the caravan. The two also agree that *they entered the defendant's caravan without any elements of coercion*, and that they *continued kissing and “making out” inside the caravan*.”  “She described that she had several “frozen moments”. *She therefore did not shout for help or try to attract attention in any other way, not even when the defendant's friend opened the door to the caravan.*” | | 2, 4, 5 |  |
| **LH-2019-119406** | “Both the defendant and the complainant have explained that after *the complainant entered the defendant's room*, they talked for quite a while.”  “*The defendant then suggests that she can lie in bed with him, which she does*.”  “The complainant explained that *the defendant stroked her back, which she said was fine*.” | | 2, 5 |  |
| *Note. Dates and locations have been removed from the texts, and the parts of the text excerpts that are especially relevant to the coding have been marked in italic.* | | | |  |
|  |  |  |  |  |
