# Supplementary material for: She Asked for It? Descriptions of Victims' Behaviors Are Associated With Sentencing in Norwegian Rape Trials
Source: Scand J Psychol. 2024 Dec 15;66(3):327–37. doi: 10.1111/sjop.13088 (PMC12042729; doi:10.1111/sjop.13088)
Supplement: Supplementary file 3 — Table S3. Dataset containing 51 court decisions used in the second control group. [file SJOP-66-327-s002.docx]

| **Table S3** |  |  |  |  |  |  |  |  |  |  |  |  |  |  |
| --- | --- | --- | --- | --- | --- | --- | --- | --- | --- | --- | --- | --- | --- | --- |
| *Dataset containing 51 court decisions used in the second control group* | | | | | | | | | | | | | | |
| **Court decision** | **IRMAS-SAFI^a^** | **Type of rape ^b^** | **Age Defendant** | **Penalty Prison** | **Penalty NOK** | **Year Conviction** | **Witnesses** | **Med Evidence ^a^** | **SMSor PhCa ^a^** | **Degree Violence ^c^** | **Place of rape ^d^** | **Photo or Video ^a^** | **Female Judges (%)** | **Nr. Items IRMAS** |
| LB-2012-116925 | 0 | 4 | 24 | 39 | 150000 | 2013 | 5 | 1 | 0 | 2 | 1 | 0 | 28 | 0 |
| LB-2013-95813 | 0 | 2 | 47 | 66 | 150000 | 2014 | 10 | 0 | 0 | 2 | 2 | 0 | 57 | 0 |
| LH-2022-104561 | 0 | 3 | 45 | 3 | 60000 | 2022 | 7 | 0 | 1 | 1 | 1 | 0 | 43 | 0 |
| LB-2015-93709 | 0 | 4 | 38 | 72 | 175000 | 2022 | 6 | 0 | 0 | 3 | 2 | 0 | 28 | 0 |
| LB-2021-132544 | 0 | 4 | 32 | 60 | 200000 | 2022 | 7 | 0 | 3 | 3 | 3 | 0 | 43 | 0 |
| LA-2016-103694 | 0 | 3 | 48 | 33 | 150000 | 2017 |  | 0 | 0 | 3 | 4 | 0 | 43 | 0 |
| LB-2014-85746 | 0 | 4 | 32 | 36 | 150000 | 2014 | 3 | 1 | 0 | 2 | 2 | 0 | 57 | 0 |
| LB-2015-65184 | 0 | 2 | 32 | 6 | 150000 | 2015 | 8 | 0 | 0 | 1 | 1 | 0 | 57 | 0 |
| LB-2016-82915 | 0 | 3 | 39 | 29 | 150000 | 2016 | 3 | 0 | 0 | 1 | 4 | 0 | 43 | 0 |
| LA-2015-50494 | 0 | 2 | 24 | 48 | 150000 | 2015 | 7 | 1 | 0 | 1 | 4 | 0 | 43 | 0 |
| LB-2013-73659 | 0 | 1 | 41 | 36 | 125000 | 2013 | 6 | 0 | 0 | 1 | 4 | 0 | 28 |  |
| LG-2020-104834 | 0 | 2 | 25 | 48 | 150000 | 2020 | 10 | 0 | 1 | 1 | 4 | 0 | 57 | 0 |
| LA-2019-112567 | 0 | 4 | 22 | 96 | 250000 | 2019 | 2 | 1 | 0 | 2 | 2 | 0 | 57 | 0 |
| LB-2017-71671 | 0 | 1 | 27 | 0 | 0 | 2018 | 8 | 0 | 0 | 1 | 1 | 0 | 33 | 0 |
| LF-2016-183090 | 0 | 1 | 28 | 2,5 | 40000 | 2017 | 2 | 1 | 0 | 1 | 4 | 0 | 57 | 0 |
| LA-2015-66242 | 0 | 4 | 27 | 63 | 175000 | 2015 |  | 1 | 0 | 2 | 2 | 0 | 43 | 0 |
| LH-2019-85632 | 0 | 2 | 27 | 0 | 0 | 2019 | 5 | 1 | 0 | 1 | 4 | 0 | 43 | 0 |
| LF-2016-51393 | 0 | 1 | 30 | 2 | 0 | 2016 | 4 | 0 | 0 | 1 | 4 | 0 | 28 | 0 |
| LG-2013-146425 | 0 | 4 | 23 | 39 | 150000 | 2014 | 8 | 0 | 0 | 2 | 2 | 0 | 28 | 0 |
| LG-2011-206540 | 0 | 4 | 30 | 48 | 150000 | 2013 | 12 | 0 | 0 | 1 | 1 | 0 | 43 | 0 |
| LF-2019-17624 | 0 | 4 | 20 | 60 | 150000 | 2019 | 2 | 0 | 0 | 2 | 2 | 0 | 57 | 0 |
| LG-2013-46486 | 0 | 4 | 28 | 45 | 175000 | 2013 | 5 | 1 | 0 | 2 | 2 | 0 | 43 | 0 |
| LG-2013-177121-2 | 0 | 3 | 24 | 42 | 150000 | 2013 | 5 | 0 | 0 | 2 | 2 | 0 | 28 | 0 |
| LA-2017-165588 | 0 | 2 | 54 | 45 | 150000 | 2017 | 6 | 1 | 0 | 1 | 3 | 0 | 43 | 0 |
| LH-2018-137483 | 0 | 2 | 24 | 48 | 150000 | 2018 | 7 | 0 | 0 | 1 | 1 | 0 | 71 | 0 |
| LA-2019-176519 | 0 | 2 | 27 | 48 | 150000 | 2020 | 9 | 1 | 0 | 1 | 4 | 0 | 28 | 0 |
| LA-2015-152142 | 0 | 4 | 36 | 60 | 150000 | 2016 |  | 0 | 0 | 2 | 2 | 0 | 43 | 0 |
| LB-2013-90520 | 0 | 3 | 31 | 48 | 150000 | 2013 |  | 0 | 0 | 2 | 2 | 0 | 57 | 0 |
| LA-2021-158204 | 0 | 3 | 45 | 0 | 150000 | 2022 | 8 | 1 | 0 | 2 | 2 | 0 | 71 | 0 |
| LA-2020-141719 | 0 | 2 | 22 | 0 | 0 | 2021 |  | 0 | 0 | 1 | 3 | 0 | 71 | 0 |
| LB-2019-171089 | 0 | 2 | 24 | 0 | 120000 | 2020 | 6 | 1 | 0 | 1 | 3 | 0 | 28 | 0 |
| LB-2017-57220 | 0 | 3 | 41 | 54 | 140000 | 2017 | 9 | 1 | 0 | 2 | 2 | 0 | 43 | 0 |
| LF-2015-117661 | 0 | 3 | 34 | 48 | 150000 | 2015 | 4 | 0 | 0 | 2 | 4 | 1 | 28 | 0 |
| LA-2017-34918 | 0 | 2 | 37 | 0 | 150000 | 2017 | 11 | 1 | 0 | 1 | 1 | 0 | 28 | 0 |
| LB-2015-90512 | 0 | 1 | 31 | 34 | 120000 | 2016 | 8 | 0 | 0 | 1 | 4 | 0 | 33 | 0 |
| LB-2013-47386 – RG | 0 | 1 | 24 | 0 | 10000 | 2013 |  | 0 | 0 | 1 | 4 | 0 | 28 | 0 |
| LF-2018-76465 | 0 | 3 | 71 | 6 | 50000 | 2018 | 2 | 0 | 0 | 1 | 4 | 0 | 43 | 0 |
| LH-2020-121065 | 0 | 1 | 32 | 7 | 70000 | 2021 | 6 | 1 | 0 | 1 | 1 | 0 | 43 | 0 |
| LB-2016-94306 | 0 | 3 | 28 | 48 | 150000 | 2017 | 7 | 0 | 0 | 2 | 4 | 0 | 28 | 0 |
| LA-2017-51710 | 0 | 4 | 23 | 12 | 90000 | 2017 |  | 0 | 0 | 1 | 4 | 0 | 43 | 0 |
| LG-2014-59605 | 0 | 1 | 45 | 36 | 125000 | 2015 | 8 | 0 | 0 | 1 | 3 | 0 | 43 | 0 |
| LG-2022-69099 | 0 | 1 | 46 | 39 | 175000 | 2022 | 5 | 0 | 0 | 1 | 1 | 0 | 43 | 0 |
| LF-2021-27521 | 0 | 2 | 31 | 45 | 150000 | 2021 | 5 | 1 | 0 | 1 | 4 | 1 | 57 | 0 |
| LB-2022-65539 | 0 | 1 | 31 | 29 | 100000 | 2022 | 9 | 1 | 0 | 1 | 4 | 0 | 43 | 0 |
| LB-2012-167114-1 | 0 | 1 | 28 | 12 | 150000 | 2013 | 1 | 0 | 0 | 1 | 3 | 0 | 28 | 0 |
| LB-2018-177307 | 0 | 2 | 52 | 48 | 150000 | 2019 |  | 0 | 0 | 1 | 4 | 0 | 43 | 0 |
| LG-2023-15188 | 0 | 4 | 45 | 44 | 250000 | 2023 | 10 | 1 | 0 | 1 | 4 | 0 | 28 | 0 |
| LB-2016-100370 | 0 | 1 | 43 | 30 | 100000 | 2017 | 11 | 0 | 0 | 1 | 4 | 0 | 28 | 0 |
| LA-2016-109675 | 0 | 2 | 51 | 39 | 120000 | 2017 |  | 1 | 1 | 1 | 1 | 0 | 43 | 0 |
| LB-2014-67463 | 0 | 1 | 34 | 30 | 120000 | 2014 | 5 | 1 | 0 | 2 | 3 | 0 | 57 | 0 |
| LH-2018-191265 | 0 | 2 | 21 | 21 | 90000 | 2019 | 6 | 1 | 0 | 1 | 2 | 1 | 57 | 0 |
| *Note. ^a^ 1 = Yes, 0 = No. ^b^ 1 = Incapacitated rape, not intercourse, 2 = Incapacitated rape, intercourse, 3 = Rape by force, not intercourse, 4 = Rape by force, intercourse. ^c^ 1 = No additional violence, 2 = Physical force, 3 = Excessive violence. ^d^ 1 = Home, 2 = Outside or in public, 3 = Defendants’ home, 4 = Other.* | | | | | | | | | | | | | | |
